# Methylation and expression of the tumour suppressor, *PRDM5*, in colorectal cancer and polyp subgroups

**DOI:** 10.1186/s12885-015-1011-9

**Published:** 2015-01-23

**Authors:** Catherine E Bond, Mark L Bettington, Sally-Ann Pearson, Diane M McKeone, Barbara A Leggett, Vicki LJ Whitehall

**Affiliations:** 1 Conjoint Gastroenterology Laboratory, QIMR Berghofer Medical Research Institute, Brisbane, Queensland Australia; 2 School of Medicine, University of Queensland, Brisbane, Queensland Australia; 3 Envoi Specialist Pathologists, Brisbane, Queensland Australia; 4 Royal Brisbane and Women’s Hospital, Brisbane, Queensland Australia; 5 Pathology Queensland, Brisbane, Queensland Australia

**Keywords:** *PRDM5*, Colorectal cancer, *BRAF* V600E mutation

## Abstract

**Background:**

*PRDM5* is an epigenetic regulator that has been recognized as an important tumour suppressor gene. Silencing of PRDM5 by promoter hypermethylation has been demonstrated in several cancer types and *PRDM5* loss results in upregulation of the Wnt pathway and increased cellular proliferation. *PRDM5* has not been extensively investigated in specific subtypes of colorectal cancers. We hypothesized it would be more commonly methylated and inactivated in serrated pathway colorectal cancers that are hallmarked by a *BRAF* V600E mutation and a methylator phenotype, compared to traditional pathway cancers that are *BRAF* wild type.

**Methods:**

Cancer (214 *BRAF* mutant, 122 *BRAF* wild type) and polyp (59 serrated polyps, 40 conventional adenomas) cohorts were analysed for *PRDM5* promoter methylation using MethyLight technology. *PRDM5* protein expression was assessed by immunohistochemistry in cancers and polyps. Mutation of *PRDM5* was analysed using cBioPortal’s publicly available database.

**Results:**

*BRAF* mutant cancers had significantly more frequent *PRDM5* promoter methylation than *BRAF* wild type cancers (77/214,36% vs 4/122,3%; p<0.0001). Serrated type polyps had a lower methylation rate than cancers but were more commonly methylated than conventional adenomas (6/59,10% vs 0/40,0%). *PRDM5* methylation was associated with advanced stages of presentation (p<0.05) and the methylator phenotype (p=0.03). PRDM5 protein expression was substantially down-regulated in both *BRAF* mutant and wild type cancer cohorts (92/97,95% and 39/44,89%). The polyp subgroups showed less silencing than the cancers, but similar rates were found between the serrated and conventional polyp cohorts (29/59, 49%; 23/40, 58% respectively). Of 295 colorectal cancers, *PRDM5* was mutated in only 6 (2%) cancers which were all *BRAF* wild type.

**Conclusions:**

Serrated pathway colorectal cancers demonstrated early and progressive *PRDM5* methylation with advancing disease. Interestingly, PRDM5 protein expression was substantially reduced in all polyp types and more so in cancers which also indicates early and increasing *PRDM5* down-regulation with disease progression. Methylation may be contributing to gene silencing in a proportion of *BRAF* mutant cancers, but the large extent of absent protein expression indicates other mechanisms are also responsible for this. These data suggest that *PRDM5* is a relevant tumour suppressor gene that is frequently targeted in colorectal tumourigenesis.

**Electronic supplementary material:**

The online version of this article (doi:10.1186/s12885-015-1011-9) contains supplementary material, which is available to authorized users.

## Background

PR (PRDI-BF1 and RIZ) domain (PRDM) proteins are a family of zinc finger transcription factors whose PR domain shares homology to the SET domain that is often present in proteins with chromatin modifying activity [1]. *PRDM5* is an epigenetic regulator that does not possess this specific activity itself, however its 16 zinc fingers facilitates sequence specific protein and DNA interactions with a multitude of genes including histone methyltransferases and deacetylases [2-4]. *PRDM5* recruits and directs these, specifically G9A and HDAC1, towards the promoters of its target genes to cause repression via chromatin modification [3]. *PRDM5* has also been found to activate genes by maintaining RNA polymerase II at its target’s promoters [5]. Loss of *PRDM5* is associated with bone morphogenic and developmental defects [5,6], and infrequent mutations of *PRDM5* have been found in brittle cornea syndrome and neutropenia [3,7].

Studies have shown its promoter region contains a CpG island that is epigenetically silenced by methylation in several different cancer cell lines and primary cancers including breast, liver, gastric, lung, nasopharyngeal and esophageal [2,4,8,9]. Functional studies have identified *PRDM5* as a tumour suppressor gene due to its role in suppressing cell growth and proliferation [4,8], in regulation of the cell cycle at the G2/M checkpoint [2,3] and as a heat shock responsive gene [8]. Furthermore, *PRDM5* has been associated with inhibition of the Wnt pathway [6], where its overexpression prevented TCF/beta-catenin dependent transcription and repressed the downstream Wnt target, CDK4 in cancer cell lines [8]. Additionally, *PRDM5* loss resulted in increased adenoma burden in mice models that had a deregulated Wnt pathway background [10].

Despite several cancers identified as having frequent *PRDM5* promoter methylation, only minimal rates of methylated *PRDM5* has been found in an uncharacterized series of colorectal cancers [4]. In a specific subgroup of colorectal cancers, there is frequent widespread methylation of promoter regions and subsequent silencing of key tumour suppressor genes, which is termed the CpG Island Methylator Phenotype (CIMP) [11,12]. These cancers derive from serrated type precursor lesions and are hallmarked by a V600E *BRAF* mutation, which with the onset of CIMP are early events in this ‘serrated pathway’ of tumourigenesis [13]. Cancers that follow the serrated pathway account for approximately 15% of all colorectal cancers. Approximately half of these cancers methylate a DNA mismatch repair gene, *MLH1*, and develop microsatellite instability (MSI) [14,15], and the remaining half stay as microsatellite stable (MSS).

The most common form of colorectal cancer originates from a conventional adenoma. These follow a ‘traditional pathway’ in which key molecular events, such as mutations of *APC* and *KRAS,* have been previously well defined [16] and result in cancers that are *BRAF* wild type and microsatellite stable.

This study has investigated whether *PRDM5* methylation is a target of epigenetic silencing more commonly in the serrated compared to the traditional pathway of colorectal cancer. This was examined in both cancer and precursor lesion subgroups to give an indication of when *PRDM5* is downregulated in tumourigenesis. *PRDM5* protein expression was also examined in cancer and polyp subgroups, and *PRDM5* mutation frequency was investigated using a publicly available database.

## Methods

### Patient samples

A total of 214 *BRAF* mutant (120 *BRAF* mutant/MSI and 94 *BRAF* mutant/MSS) and 122 *BRAF* wild type cancers were obtained either as fresh frozen tissue after surgical excision from the Royal Brisbane and Women’s Hospital (RBWH), Brisbane, Australia as previously described [17,18], or as formalin-fixed paraffin embedded (FFPE) tissue from Envoi Specialist Pathologists, Brisbane, Australia. Written, informed consent was obtained from each patient involved in this research which was approved by the Royal Brisbane and Women’s Hospital and Bancroft Human Research Ethics Committee. Clinicopathological data of patient gender, age at diagnosis, anatomical site of cancer (with proximal termed if proximal to the splenic flexure), and cancer stage (according to the American Joint Committee on Cancer, AJCC, system) were collected where available.

Polyp cohorts consisting of 59 serrated type polyps (19 microvesicular hyperplastic polyps, MVHPs; 20 sessile serrated adenomas, SSAs; and 20 traditional serrated adenomas, TSAs) and 40 conventional polyps (20 of each tubular adenomas, TAs; and tubulovillous adenomas, TVAs) were collected as FFPE tissue from Envoi Specialist Pathologists.

DNA from fresh cancer and matched normal tissue was extracted using AllPrep DNA mini kit (Qiagen, Dusseldorf, Germany). DNA from the FFPE cancer and polyp and matched normals were extracted by the Chelex-100 method (Bio-Rad Laboratories, CA, USA).

The presence of MSI had been previously analysed for the RBWH’s cancer samples using the National Cancer Institute’s 5 marker panel [17,19]. Cancers from Envoi Pathologists were evaluated for immunohistochemical loss of mismatch repair protein expression (*MLH1*, *PMS2*, *MSH6*, *MSH2*) as a surrogate for MSI. Presence of the *BRAF* V600E (a1796t) mutation, *p53* mutation (over exons 4–8) and *KRAS* mutation (over codons 2 and 3) had been previously investigated for the RBWH’s samples [17]; presence of *BRAF* V600E (a1796t) and *KRAS* (codons 2 and 3) mutations was analysed for Envoi’s samples as previously described [17,20-22].

### CpG Island Methylator Phenotype (CIMP) analysis and *PRDM5* Methylation-Specific PCR

Sample DNA was bisulfite modified using Epitect Fast Bisulfite Conversion kit (Qiagen, Dusseldorf, Germany). CIMP was assessed in cancer and polyp cohorts using MethyLight technology over a 5 marker panel consisting of *CACNA1G*, *IGF2*, *NEUROG1*, *RUNX3* and *SOCS1* as previously described by Weisenberger et al. [17,22-24]. Percent of methylated reference (PMR) indicates the extent of methylation of a sample in relation to a methylated reference, and a sample with a PMR of ≥10 was considered as methylated at that marker [22]. If ≥3 markers were methylated the sample was considered CIMP-high, with 1–3 markers methylated the sample was termed CIMP-low and CIMP-0 if no markers were methylated [22]. For MSP of *PRDM5*, the same PMR cutoff of ≥10 applied for a sample to be considered methylated, a sample with a PMR <10 was considered unmethylated. For all CIMP markers and the *PRDM5* MSPs, an Alu assay was included for each sample as a measure of the success of bisulfite conversion of that sample [22]. A cycle threshold for Alu of <23 was the sample inclusion criteria [25,26]. *PRDM5* is on the reverse strand and the primer and probe sequences are as follows:

- F: 5′AAAACTAAACAAAAACGAAAACGCA; R: 5′GGTTTTAAATTCGGAGGTTCGC;
- Probe: 5′ 6FAM-CGCGCCGAAACTAAAAATACTAACG–BHQ1.

### *PRDM5* and beta catenin immunohistochemistry

Tissue sections were obtained from formalin-fixed paraffin embedded (FFPE) blocks. Antigen retrieval was performed at low pH (pH6, Reveal decloaker; Biocare Medical, CA, USA) for 15mins at 105 °C. H_2_O_2_ and Sniper were used to facilitate endogenous peroxidase and protein blocks respectively. PRDM5 antibody (anti-PRDM5, LS-1982, Lifespan BioSciences, Seattle, USA) was manually applied at 1/750 dilution and left for 1 hour. MACH3 Rabbit secondary antibody probe and polymer was applied for 10 and 20 minutes respectively (Biocare Medical, CA, USA), and DAB chromagen (Biocare Medical, CA, USA) was applied for 5 minutes. Beta-catenin antibody (anti-Beta-catenin 224 M16 (14) Cell Marque, California, USA) was manually applied and left for 1.5 hrs. MACH1 Rabbit secondary antibody probe and polymer was applied for 15 and 30 minutes respectively, and DAB was applied for 8 minutes. Sections were counterstained with haematoxylin. Slides were examined by an expert gastrointestinal pathologist and scored as either positive or negative depending on presence or absence of *PRDM5* staining, and presence of nuclear beta-catenin was observed and scored either positive or negative accordingly.

### Statistical analysis

Significant differences between categorical data were analysed with Fisher‘s exact test or Pearson’s chi-squared test where appropriate. Significance between continuous data was analysed by a student’s t-test. P values <0.05 were considered significant.

## Results

### Clinical and molecular findings of cancer cohorts

Clinical and molecular differences between the *BRAF* mutant and *BRAF* wild type cohorts concurred with previous findings [17,18]. The *BRAF* mutant cancers had an older age of onset, a propensity to affect females, a frequent proximal tumour location, an earlier stage at presentation and a mucinous histology compared to *BRAF* wild type cancers (Table 1). As expected, *BRAF* mutant cancers were predominantly CIMP high and had a lower rate of *p53* mutation compared to *BRAF* wild type cancers (Table 1).

**Table 1:** **Clinical and molecular features of cancer cohorts**

	*BRAF*mutant	*BRAF*wild type	P value
N	214	122	-
Average Age (years)	74.1	67.1	**<0.0001**
Gender - female	139/214 (65.0%)	49/122 (40.2%)	**<0.0001**
Tumour Location (Proximal)	164/192 (85.4%)	28/117 (23.9%)	**<0.0001**
AJCC stage I/II	110/170 (64.7%)	58/111 (52.3%)	**<0.05**
AJCC stage III/IV	60/170 (35.3%)	53/111 (47.7%)	
Mucinous	37/96 (38.5%)	3/42 (7.1%)	**<0.0001**
Differentiation (poor)	38/96 (39.6%)	12/42 (28.6%)	0.2
MSI High	120/214 (56.1%)	0	-
CIMP High	154/205 (75.1%)	3/121 (2.5%)	**<0.0001**
*p53* Mutation	29/107 (27.1%)	40/80 (50.0%)	**0.002**
*KRAS* Mutation	0	38/80 (47.5%)	**-**
*PRDM5* Methylation	77/214 (36.0%)	4/122 (3.3%)	**<0.0001**
*PRDM5* PMR	27	3	**<0.0001**
Nuclear Beta-Catenin	36/92 (39.1%)	36/42 (85.7%)	**<0.0001**

Significant p values indicated in bold text.

The *BRAF* mutant cohort was comprised of 120 MSI (56.1%) and 94 MSS (43.9%) cancers (Table 1). When clinical and molecular parameters were considered within this cohort with microsatellite status considered, the differences again correlated with previous findings [17,18]. *BRAF* mutant/MSS cancers affected patients at a younger average age, more frequently presented at advanced stages and were less commonly proximally located than *BRAF* mutant/MSI cancers (Additional file 1: Table S1). Additionally, *BRAF* mutant/MSS cancers were not as frequently CIMP high, but were more frequently *p53* mutant compared to *BRAF* mutant/MSI cancers (Additional file 1: Table S1).

### *PRDM5* methylation in cancer cohorts

*PRDM5* was methylated in 77/214 (36.0%) *BRAF* mutant cancers compared to 4/122 (3.3%) *BRAF* wild type cancers (p < 0.0001). Similarly, the average percentage of methylated reference (PMR) scores which indicates the extent of methylation of a cancer relative to a methylase treated reference sample, was significantly higher in the *BRAF* mutant compared to the *BRAF* wild type cohort (27 vs 3; p < 0.0001) (Table 1). There was no significant difference in *PRDM5* methylation rates within the *BRAF* mutant cohort when stratified for microsatellite status (Additional file 1: Table S1).

*BRAF* mutant cancers that had methylated *PRDM5* were more likely to present at advanced stages compared to *BRAF* mutant cancers with unmethylated *PRDM5* (AJCC stage III/IV: 29/65, 44.6% vs 31/105, 29.5%; p < 0.05) (Table 2). *PRDM5* methylation correlated with CIMP high. CIMP high was strongly prevalent in the *BRAF* mutant/MSI cancers (at 86%), therefore this was evident in the *BRAF* mutant/MSS cohort (61% CIMP high rate) where CIMP high was more frequent in *PRDM5* methylated compared to unmethylated cancers (27/36, 75.0% vs 27/53 50.9%; p = 0.03) (Additional file 1: Table S2).

**Table 2:** **Comparison of clinical and molecular features of** ***BRAF*** **mutant cancers stratified by** ***PRDM5*** **methylation status (n = 214)**

	*PRDM5*Methylated	*PRDM5*Unmethylated	P value
N *BRAF* mutant cancers	77/214 (36.0%)	137/214 (64.0%)	
Average Age	72.7	75.0	0.1
Gender (Female)	48/77 (62.3%)	91/137 (66.4%)	0.6
Location (Proximal)	62/72 (86.1%)	102/120 (84.2%)	1.0
AJCC stage I/II	36/65 (55.4%)	74/105 (70.5%)	**<0.05**
AJCC stage III/IV	29/65 (44.6%)	31/105 (29.5%)	
Mucinous	9/34 (26.5%)	28/62 (45.2%)	0.08
Differentiation (poor)	15/34 (44.1%)	23/62 (37.1%)	0.5
MSI High	37/77 (48.1%)	83/137 (60.6%)	0.09
CIMP High	59/73 (80.8%)	95/132 (72.0%)	0.2
*p53* Mutation	13/39 (33.3%)	15/68 (22.1%)	0.3
Nuclear Beta-Catenin	16/33 (48.5%)	20/59 (33.9%)	0.2

Significant p values indicated in bold text.

### PRDM5 protein expression in cancer cohorts

**Figure 1:**
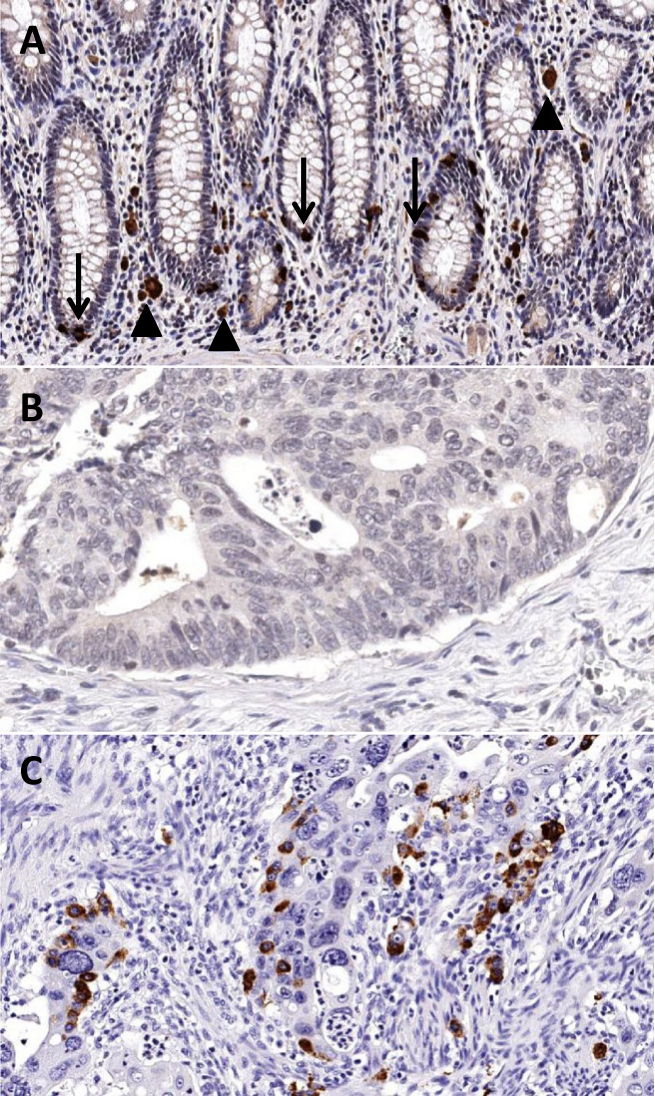
**PRDM5 immunohistochemistry. A** demonstrates the normal pattern of staining in non-neoplastic colonic mucosa. Down arrows indicate strong nuclear and cytoplasmic staining for PRDM5 in scattered cells predominantly in the crypt bases. Up arrowheads indicate incidental melanosis coli in the lamina propria. **B** is a representative area of a colorectal carcinoma negative for PRDM5. **C** is a representative area of a colorectal carcinoma positive for PRDM5, showing scattered cells with strong cytoplasmic and/or nuclear staining. (Original magnification: x200).

Immunohistochemical analysis of PRDM5 protein expression in adjacent normal mucosa showed it was routinely present within the crypt bases. Interestingly, there was substantial loss of protein expression in both the *BRAF* mutant (92/97, 94.5%) and *BRAF* wild type (39/44, 88.6%) cancer cohorts (Figure 1).

Of the 10 cancers with retained PRDM5 protein expression, 9 had unmethylated *PRDM5*. The high rate of PRDM5 protein loss compared to the rate of methylation across all cancers, and the high frequency of absent PRDM5 protein expression observed in unmethylated cancers, clearly indicates that other mechanisms besides methylation are contributing to PRDM5 protein down-regulation.

### *PRDM5* methylation and protein expression in polyp cohorts

Fifty-nine serrated type precursor lesions (19 MVHPs, 20 SSAs, 20 TSAs), and forty conventional type precursor lesions (20 TAs, 20 TVAs) were included in the analysis. Molecularly, the serrated polyps were significantly more methylated and *BRAF* mutant as expected, and all polyp subtypes had a low *KRAS* mutation rate (Table 3). Clinically, the TVAs and TSAs were more likely to be larger and distally located (Additional file 1: Table S3).

**Table 3:** ***PRDM5*** **methylation and expression and other molecular features of serrated and conventional polyp subgroups**

	Serrated Polyps			Conventional Adenomas		P value
	MVHP	SSA	TSA	TVA	TA	Serrated vs conventional
N	19	20	20	20	20	-
***PRDM5*** **Methylation**	2 (11%)	2 (10%)	2 (10%)	0	0	0.08
***PRDM5*** **Average**	3.4	2.2	3.6	1.1	0.4	**0.01**
**PMR score**						
**PRDM5 IHC**	5 (26%)	12 (60%)	12 (60%)	12 (60%)	11 (55%)	0.5
**Negative Expression**						
*BRAF* mutation	18 (95%)	17 (85%)	13 (65%)	0	0	**<0.0001**
*KRAS* mutation	0	1 (5%)	4 (20%)	3 (15%)	1 (5%)	1.0
CIMP High	2 (11%)	12 (60%)	9 (45%)	0	0	**<0.0001**
Nuclear Beta-Catenin	0	0	3 (15%)	17 (85%)	9 (45%)	**<0.0001**

Significant p values indicated in bold text.

Methylation and protein expression of *PRDM5* was analysed across all polyp subgroups to determine whether down-regulated *PRDM5* was an early event in tumourigenesis and in which polyp type or pathway this was mostly occurring in.

*PRDM5* was methylated in 2 of each of the three serrated polyp subtypes to give an overall methylation rate of 10% (6/59) in the serrated polyps compared to the lack of methylation in both subtypes of conventional polyps (0/40) (p = 0.08). The average PMR of methylated *PRDM5* was significantly higher in all serrated polyp subtypes compared to the conventional polyps (p = 0.01) (Table 3).

PRDM5 protein expression was reduced across the serrated SSAs and TSAs, and conventional TAs and TVAs polyp subgroups at similar frequencies (an average of 59%). MVHPs which are the earliest form of serrated lesion, had a lower rate of loss (at 26%) compared to SSAs (60%) and TSAs (45%), which suggests there is a progressive down-regulation of *PRDM5* with advancing disease (Table 3). Due to the greater rates of PRDM5 protein loss compared to methylation, other mechanisms are contributing to this silencing especially in the conventional polyps, as was seen in the cancer cohorts.

As expected, the rate of *BRAF* V600E mutation and CIMP high was significantly more common in serrated type polyps than conventional polyps (both p < 0.0001), and *KRAS* mutation in codons 2 and 3 was relatively low across both serrated and conventional polyp types (8% and 10% respectively) (Table 3).

### *PRDM5* mutation analysis

A publicly available database was searched for presence of *PRDM5* mutations in colorectal cancer. cBioPortal (www.cBioPortal.org) [27] incorporates data from The Cancer Genome Atlas Network’s colorectal cancer study [28] and Seshagiri *et al.* 2010 [29]. Collectively there are 296 colorectal cancers with somatic mutation data that show an 8.1% *BRAF* V600E mutation rate. In total, 7 *PRDM5* mutations were reported from 6 cancer samples (2.0% mutation rate) that were all *BRAF* wild type. These mutations were spread along the length of the gene and no two were similar. Due to the low rate of *PRDM5* mutation found, this type of analysis was not extended to this study’s cancer or polyp cohorts.

### Presence of nuclear beta-catenin in cancer and polyp subgroups

Nuclear beta-catenin as a surrogate of Wnt pathway activation was present in 39% (36/92) *BRAF* mutant cancers which was significantly lower than the rate observed in *BRAF* wild type cancers at 86% (36/42) (p < 0.0001) (Table 1). As expected, there was also a significantly reduced rate of nuclear beta-catenin in serrated compared to conventional polyps (3/59, 5% vs 26/40, 65%) (p < 0.0001) (Table 3).

Due to previous correlations of methylated *PRDM5* with presence of active beta-catenin and exogenous *PRDM5* causing a decrease in downstream Wnt reporter assays [8], presence of methylated *PRDM5* and nuclear beta-catenin was assessed within the cancer cohorts.

There was no significant association of nuclear beta-catenin occurring in *PRDM5* methylated compared to unmethylated *BRAF* mutant cancers (16/33; 49% vs 20/59, 34%) (p = 0.2) (Table 2). Only when stratified for MSI status, was a correlation observed with a higher rate of nuclear beta-catenin in *PRDM5* methylated compared to unmethylated *BRAF* mutant/MSI cancers (13/21, 62% vs 12/38, 32%) (p = 0.03) (Additional file 1: Table S2). No correlation with presence of nuclear beta-catenin and methylated *PRDM5* was seen in *BRAF* mutant/MSS cancers.

## Discussion

This study investigated a large series of molecularly subtyped colorectal cancers and precursor lesions for the presence of *PRDM5* methylation and protein expression. We found the *PRDM5* promoter region was substantially methylated in *BRAF* mutant cancers of the serrated pathway whereas minimal levels of methylation were detected in the *BRAF* wild type cancers of the traditional pathway. This is the first study to show that a particular subgroup of colorectal cancer has a comparably high rate of *PRDM5* methylation as previously found in other cancers such as lung, breast, liver and gastric cancer [2,4,8,9].

*PRDM5* methylation was evident in a small proportion of serrated type polyps which indicates this may be an early event in tumourigenesis in the serrated pathway. The frequency of *BRAF* mutation and CIMP increased from serrated polyp to cancer as expected. The frequency of *PRDM5* methylation also increased from serrated precursor lesion to *BRAF* mutant cancers at a similar proportion which suggests that *PRDM5* methylation associates with advancing disease in cancers of the serrated pathway. Furthermore, there was an association of *PRDM5* methylation being more prevalent in *BRAF* mutant cancers presenting at late compared to early stages which further indicates epigenetic regulation of *PRDM5* may influence disease progression in the serrated pathway. This association was also seen in a previous study where *PRDM5* methylation was more common in high grade breast and liver cancers [2].

The absence of *PRDM5* methylation found in conventional adenomas and the low rate seen in *BRAF* wild type cancers indicates that *PRDM5* methylation is not an important event in traditional pathway cancers. This minimal *PRDM5* methylation rate in *BRAF* wild type cancers, at 3%, was similar to the low frequency found in the one other study that investigated primary colorectal cancers [4], and others have concluded that there is only a negligible rate of *PRDM5* methylation in colorectal cancer based on cell line analysis [8]. The findings from this study highlights the importance of stratifying for molecular subtype with analysis of molecular markers involved in colorectal cancer as it is a heterogenous disease comprised of several clinically and genetically distinct subtypes. Although the *KRAS* mutation rate was minimal in the conventional adenoma cohorts, similarly low rates, particularly for TAs, have been previously found [20,30], and wide variations of *KRAS* mutation rates in adenomas have been reported [31-33].

CIMP is highly prevalent in cancers of the serrated pathway, particularly those that are microsatellite unstable. When the *BRAF* mutant cancers were stratified for MSI status, it was apparent that *PRDM5* methylation correlated with CIMP. However, it is unlikely that *PRDM5* methylation is merely a passenger event of CIMP. This is due to there being a considerable presence of *PRDM5* methylation and transcript down-regulation in several non-CIMP cancer types [2,4,8,9], and there is a lack of reported *PRDM5* methylation in other CIMP related cancers such as glioma. Additionally, the substantial loss of *PRDM5* protein expression found in this study, suggests that potentially methylation and loss of *PRDM5* is highly relevant in tumourigenesis [34].

Endogenous *PRDM5* protein expression was routinely detected in normal tissue sections in this study which concurs with a previous investigation that found *PRDM5* transcript expression was prevalent in several normal tissues [8]. Interestingly the vast majority of cancers in both the *BRAF* mutant and *BRAF* wild type cancer cohorts lacked *PRDM5* protein expression. Absent expression was also widespread in both serrated and conventional polyps, although this rate of downregulation was less than that in the cancers. MVHPs which are the earliest form of serrated lesion and may give rise to SSAs, had the least frequency of absent *PRDM5* protein expression, and overall this analysis demonstrates an early and linear progression of downregulated *PRDM5* with advancing disease across all colorectal subgroups of both the serrated and traditional pathways.

This frequent loss of PRDM5 protein expression seen by immunohistochemistry is concordant with findings of a previous study that investigated expression in 18 colorectal cancers that were not molecularly subtyped [10]. However, half of this study’s normal sections had no observed endogenous protein which may indicate the inability of the antibody used to reliably detect protein within their cancer samples. Of the 10 cancers in the present study that retained PRDM5 protein expression, there was 90% concordance with these cancers being unmethylated. The one cancer that was methylated but still expressed PRDM5 protein may be in the seeding stages of methylation, and although the relatively few CpG sites covered by the methylight assay were methylated, they were not sufficient to fully silence protein expression. Additionally, the cancers that were methylated with concordant absent expression, may represent the presence of a more global methylation pattern driving protein loss in these cancers. Similar incidences of methylated gastric and esophageal cancer cell lines showing positive transcript expression has been observed [8]. This study’s methylight assay was in very close proximity to the MSP of Shu et al’s [8] in the promoter region which helps to further suggest that in some cancers, extensive methylation over the promoter is required for complete down-regulation.

Previous studies have mostly analysed *PRDM5* transcript expression as a measure of the extent of silencing [2,4,8,35]. Although one reported similar findings to this current study where decreased transcript expression was observed in unmethylated gastric cancers [4], concordance was found between reduced transcript expression and methylation of nasopharyngeal cancers, and therefore silencing induced primarily by methylation was concluded [8]. This current study analysed protein expression which is a more relevant determinant of the functional endpoint state of the gene and it reflects any post translational modifications that may have taken place. Results showed a far greater rate of loss compared to methylation frequency, indicating that in colorectal cancer methylation is just one of the mechanisms responsible for this.

*PRDM5* mutation events contribute to brittle cornea syndrome and neutropenia [3,7], however they have not been analysed in cancer types previously. This study utilised a publicly available database, cBioPortal [27], which incorporates data from two large series of colorectal cancers [28,29]. Overall a low rate of mutation was found and there was no identifiable mutational hotspot which indicated this mechanism is not a common cause of down-regulation. However, all mutations were present in *BRAF* wild type cancers which may still indicate this mechanism is of some relevance in traditional pathway cancers.

*PRDM5* is located on chromosome 4q27 which is within a region commonly deleted in colorectal cancer [36,37]. Analysis of recent SNP array data, revealed loss over this locus in 33% *BRAF* mutant/MSS and 44% *BRAF* wild type cancers [38], which suggests gene deletion may also contribute to the levels of down-regulation observed. It was found that the colorectal cancer cell line, SW480, lacked *PRDM5* expression due to methylation of histone H3K27 and not as a result of a methylated promoter region [4]. Therefore, histone modification events that can alter chromatin structure and result in gene suppression provide a further mechanism of *PRDM5* silencing. Additionally, small and long regulatory RNAs may be acting at both the post-transcriptional and pos-translational stages to effect gene and /or protein expression [39], as well as one of the many other post-translational modifications such as acetylation and that could be taking place to affect expression.

The Wnt pathway is one of the most aberrantly upregulated pathways present in colorectal cancer [28]. Previous findings have shown that *PRDM5* can interact with a variety of genes involved in inhibition of the Wnt pathway [6], *PRDM5* loss results in an increased number of intestinal adenomas on an upregulated Wnt background [10], and methylated *PRDM5* has been correlated with presence of active beta-catenin in cancer cell lines [8]. In this study, methylated *PRDM5* associated with presence of nuclear beta-catenin in the *BRAF* mutant/MSI cancers (Additional file 1: Table S2). These cancers, through their heavily methylated phenotype have been found to methylate other inhibitors of the Wnt pathway such as *DKK1* and *AXIN2* [40,41], which indicates epigenetic regulation of the Wnt pathway may be more prevalent in the *BRAF* mutant/MSI compared to other CRC subtypes.

## Conclusions

This is the first study that has analysed the rate of *PRDM5* methylation and protein expression in a large and well characterized series of colorectal cancer and polyp subgroups.

*PRDM5* methylation was found to be an early event with progressive acquisition in *BRAF* mutant cancers of the serrated pathway. Furthermore, *PRDM5* protein levels were substantially reduced across both serrated and conventional polyp types and more so in *BRAF* mutant and wild type cancers. This indicates that down-regulation is initiated early in tumourigenesis and is progressive with disease advancement in both the serrated and traditional pathways. Epigenetic modification may be contributing to gene silencing in a proportion of *BRAF* mutant cancers and the large extent of absent protein expression indicates other mechanisms are also responsible for *PRDM5* down-regulation. *PRDM5* mutation was present in a small percentage of *BRAF* wild type cancers and this may be a cause of downregulation in this cancer subgroup. Overall, this investigation highlights *PRDM5* as an important tumour suppressor gene in colorectal cancer.

## Additional file

Additional file 1:
**Clinical and Molecular Features of Cancer Cohorts Stratified by MSI Status; Clinical and Molecular Features of** ***BRAF*** **mutant cohorts stratified by** ***PRDM5*** **Methylation Status; Clinical data for serrated polyp and conventional adenoma cohorts.**

## Abbreviations

AJCC: American Joint Committee on Cancer
CIMP: CpG Island Methylator Phenotype
CRC: Colorectal cancer
FFPE: Formalin-fixed paraffin embedded
MSI: Microsatellite instability
MSP: Methylation-specific PCR
MSS: Microsatellite stable
MVHP: Micro-vesicular hyperplastic polyp
PMR: Percent of methylated reference
PRDM: PRDI-BF1 and RIZ domain
SSA: Sessile serrated adenoma
TA: Tubular adenoma
TSA: Traditional serrated adenoma
TVA: Tubulovillous adenoma

## References

[1] Huang S, Shao G, Liu L (1998). The PR domain of the Rb-binding zinc finger protein RIZ1 is a protein binding interface and is related to the SET domain functioning in chromatin-mediated gene expression. J Biol Chem.

[2] Deng Q, Huang S (2004). PRDM5 is silenced in human cancers and has growth suppressive activities. Oncogene.

[3] Duan Z, Person RE, Lee HH, Huang S, Donadieu J, Badolato R, Grimes HL, Papayannopoulou T, Horwitz MS (2007). Epigenetic regulation of protein-coding and microRNA genes by the Gfi1-interacting tumor suppressor PRDM5. Mol Cell Biol.

[4] Watanabe Y, Toyota M, Kondo Y, Suzuki H, Imai T, Ohe-Toyota M, Maruyama R, Nojima M, Sasaki Y, Sekido Y (2007). PRDM5 identified as a target of epigenetic silencing in colorectal and gastric cancer. Clin Cancer Res.

[5] Galli GG, Honnens de Lichtenberg K, Carrara M, Hans W, Wuelling M, Mentz B, Multhaupt HA, Fog CK, Jensen KT, Rappsilber J (2012). Prdm5 regulates collagen gene transcription by association with RNA polymerase II in developing bone. PLoS Genet.

[6] Meani N, Pezzimenti F, Deflorian G, Mione M, Alcalay M (2009). The tumor suppressor PRDM5 regulates Wnt signaling at early stages of zebrafish development. PLoS One.

[7] Burkitt Wright EM, Spencer HL, Daly SB, Manson FD, Zeef LA, Urquhart J, Zoppi N, Bonshek R, Tosounidis I, Mohan M (2011). Mutations in PRDM5 in brittle cornea syndrome identify a pathway regulating extracellular matrix development and maintenance. Am J Hum Genet.

[8] Shu XS, Geng H, Li L, Ying J, Ma C, Wang Y, Poon FF, Wang X, Ying Y, Yeo W (2011). The epigenetic modifier PRDM5 functions as a tumor suppressor through modulating WNT/beta-catenin signaling and is frequently silenced in multiple tumors. PLoS One.

[9] Tan SX, Hu RC, Tan YL, Liu JJ, Liu WE (2014). Promoter methylation-mediated downregulation of PRDM5 contributes to the development of lung squamous cell carcinoma. Tumour Biol.

[10] Galli GG, Multhaupt HA, Carrara M, de Lichtenberg KH, Christensen IB, Linnemann D, Santoni-Rugiu E, Calogero RA, Lund AH (2014). Prdm5 suppresses Apc-driven intestinal adenomas and regulates monoacylglycerol lipase expression. Oncogene.

[11] Toyota M, Ho C, Ahuja N, Jair KW, Li Q, Ohe-Toyota M, Baylin SB, Issa JP (1999). Identification of differentially methylated sequences in colorectal cancer by methylated CpG island amplification. Cancer Res.

[12] Toyota M, Ahuja N, Ohe-Toyota M, Herman JG, Baylin SB, Issa JP (1999). CpG island methylator phenotype in colorectal cancer. Proc Natl Acad Sci U S A.

[13] Leggett B, Whitehall V (2010). Role of the serrated pathway in colorectal cancer pathogenesis. Gastroenterology.

[14] Koinuma K, Shitoh K, Miyakura Y, Furukawa T, Yamashita Y, Ota J, Ohki R, Choi YL, Wada T, Konishi F (2004). Mutations of BRAF are associated with extensive hMLH1 promoter methylation in sporadic colorectal carcinomas. Int J Cancer.

[15] Kane MF, Loda M, Gaida GM, Lipman J, Mishra R, Goldman H, Jessup JM, Kolodner R (1997). Methylation of the hMLH1 promoter correlates with lack of expression of hMLH1 in sporadic colon tumors and mismatch repair-defective human tumor cell lines. Cancer Res.

[16] Fearon ER, Vogelstein B (1990). A genetic model for colorectal tumorigenesis. Cell.

[17] Bond CE, Umapathy A, Ramsnes I, Greco SA, Zhen Zhao Z, Mallitt KA, Buttenshaw RL, Montgomery GW, Leggett BA, Whitehall VL (2012). p53 mutation is common in microsatellite stable, BRAF mutant colorectal cancers. Int J Cancer.

[18] Bond CE, Umapathy A, Buttenshaw RL, Wockner L, Leggett BA, Whitehall VL (2012). Chromosomal instability in BRAF mutant, microsatellite stable colorectal cancers. PLoS One.

[19] Boland CR, Thibodeau SN, Hamilton SR, Sidransky D, Eshleman JR, Burt RW, Meltzer SJ, Rodriguez-Bigas MA, Fodde R, Ranzani GN (1998). A National Cancer Institute Workshop on Microsatellite Instability for cancer detection and familial predisposition: development of international criteria for the determination of microsatellite instability in colorectal cancer. Cancer Res.

[20] Spring KJ, Zhao ZZ, Karamatic R, Walsh MD, Whitehall VL, Pike T, Simms LA, Young J, James M, Montgomery GW (2006). High prevalence of sessile serrated adenomas with BRAF mutations: a prospective study of patients undergoing colonoscopy. Gastroenterology.

[21] Zhao ZZ, Nyholt DR, Le L, Martin NG, James MR, Treloar SA, Montgomery GW (2006). KRAS variation and risk of endometriosis. Mol Hum Reprod.

[22] Weisenberger DJ, Siegmund KD, Campan M, Young J, Long TI, Faasse MA, Kang GH, Widschwendter M, Weener D, Buchanan D (2006). CpG island methylator phenotype underlies sporadic microsatellite instability and is tightly associated with BRAF mutation in colorectal cancer. Nat Genet.

[23] Weisenberger DJ, Campan M, Long TI, Kim M, Woods C, Fiala E, Ehrlich M, Laird PW (2005). Analysis of repetitive element DNA methylation by MethyLight. Nucleic Acids Res.

[24] Whitehall VL, Rickman C, Bond CE, Ramsnes I, Greco SA, Umapathy A, McKeone D, Faleiro RJ, Buttenshaw RL, Worthley DL (2012). Oncogenic PIK3CA mutations in colorectal cancers and polyps. Int J Cancer.

[25] Burnett-Hartman AN, Newcomb PA, Potter JD, Passarelli MN, Phipps AI, Wurscher MA, Grady WM, Zhu LC, Upton MP, Makar KW (2013). Genomic aberrations occurring in subsets of serrated colorectal lesions but not conventional adenomas. Cancer Res.

[26] Bettington M WN, Rosty C, Brown I, Clouston A, McKeone D, Pearson S, Leggett B, Whitehall V: A clinicopathological and molecular analysis of 200 traditional serrated adenomas. Modern Pathology. 2014, in print. 10.1038/modpathol.2014.122 25216220

[27] Cerami E, Gao J, Dogrusoz U, Gross BE, Sumer SO, Aksoy BA, Jacobsen A, Byrne CJ, Heuer ML, Larsson E (2012). The cBio cancer genomics portal: an open platform for exploring multidimensional cancer genomics data. Cancer Discov.

[28] TCGA (2012). Comprehensive molecular characterization of human colon and rectal cancer. Nature.

[29] Seshagiri S, Stawiski EW, Durinck S, Modrusan Z, Storm EE, Conboy CB, Chaudhuri S, Guan Y, Janakiraman V, Jaiswal BS (2012). Recurrent R-spondin fusions in colon cancer. Nature.

[30] Fernando WC, Miranda MS, Worthley DL, Togashi K, Watters DJ, Leggett BA, Spring KJ (2014). The CIMP Phenotype in BRAF Mutant Serrated Polyps from a Prospective Colonoscopy Patient Cohort. Gastroenterol Res Pract.

[31] Kakar S, Deng G, Cun L, Sahai V, Kim YS (2008). CpG island methylation is frequently present in tubulovillous and villous adenomas and correlates with size, site, and villous component. Hum Pathol.

[32] Yagi K, Takahashi H, Akagi K, Matsusaka K, Seto Y, Aburatani H, Nakajima A, Kaneda A (2012). Intermediate methylation epigenotype and its correlation to KRAS mutation in conventional colorectal adenoma. Am J Pathol.

[33] Yadamsuren EA, Nagy S, Pajor L, Lacza A, Bogner B (2012). Characteristics of advanced- and non advanced sporadic polypoid colorectal adenomas: correlation to KRAS mutations. Pathol Oncol Res.

[34] Shen H, Laird PW (2013). Interplay between the cancer genome and epigenome. Cell.

[35] Cheng HY, Chen XW, Cheng L, Liu YD, Lou G (2010). DNA methylation and carcinogenesis of PRDM5 in cervical cancer. J Cancer Res Clin Oncol.

[36] Malkhosyan S, Yasuda J, Soto JL, Sekiya T, Yokota J, Perucho M (1998). Molecular karyotype (amplotype) of metastatic colorectal cancer by unbiased arbitrarily primed PCR DNA fingerprinting. Proc Natl Acad Sci U S A.

[37] Arribas R, Risques RA, Gonzalez-Garcia I, Masramon L, Aiza G, Ribas M, Capella G, Peinado MA (1999). Tracking recurrent quantitative genomic alterations in colorectal cancer: allelic losses in chromosome 4 correlate with tumor aggressiveness. Lab Invest.

[38] Bond CE, Nancarrow DJ, Wockner LF, Wallace L, Montgomery GW, Leggett BA, Whitehall VL (2014). Microsatellite stable colorectal cancers stratified by the BRAF V600E mutation show distinct patterns of chromosomal instability. PLoS One.

[39] Cheetham SW, Gruhl F, Mattick JS, Dinger ME (2013). Long noncoding RNAs and the genetics of cancer. Br J Cancer.

[40] Rawson JB, Manno M, Mrkonjic M, Daftary D, Dicks E, Buchanan DD, Younghusband HB, Parfrey PS, Young JP, Pollett A (2011). Promoter methylation of Wnt antagonists DKK1 and SFRP1 is associated with opposing tumor subtypes in two large populations of colorectal cancer patients. Carcinogenesis.

[41] Koinuma K, Yamashita Y, Liu W, Hatanaka H, Kurashina K, Wada T, Takada S, Kaneda R, Choi YL, Fujiwara SI (2006). Epigenetic silencing of AXIN2 in colorectal carcinoma with microsatellite instability. Oncogene.

