# Supplementary material for: Methylation and expression of the tumour suppressor, PRDM5, in colorectal cancer and polyp subgroups
Source: BMC Cancer. 2015 Jan 23;15:20. doi: 10.1186/s12885-015-1011-9 (PMC4318154; doi:10.1186/s12885-015-1011-9)
Supplement: Additional file 1: — Clinical and Molecular Features of Cancer Cohorts Stratified by MSI Status; Clinical and Molecular Features of BRAF mutant cohorts stratified by PRDM5 Methylation Status; Clinical data for serrated polyp and conventional adenoma cohorts. [file 12885_2015_1011_MOESM1_ESM.docx]

**Additional File 1 – Methylation and expression of the tumour suppressor, *PRDM5*, in colorectal cancer and polyp subgroups**

Table S1: Clinical and Molecular Features of Cancer Cohorts Stratified by MSI Status

|  | 1) *BRAF* mutant /MSI | 2) *BRAF* mutant /MSS | 3) *BRAF* wild type | P value |
| --- | --- | --- | --- | --- |
| N | 120 | 94 | 122 | - |
| Average Age (years) | 76.8 | 70.2 | 67.1 | **<0.0001** |
|  |  |  |  | 1 vs 2: <0.0001 |
|  |  |  |  | 1 vs 3: <0.0001 |
|  |  |  |  | 2 vs 3: 0.09 |
| Gender (female) | 84/120 (70.0%) | 55/94 (58.5%) | 49/122 (40.2%) | **<0.0001** |
|  |  |  |  | 1 vs 2: 0.06 |
|  |  |  |  | 1 vs 3: <0.0001 |
|  |  |  |  | 2 vs 3: 0.01 |
| Tumour Location (Prox) | 102/113 (90.3%) | 62/79 (78.5%) | 28/117 (23.9%) | **<0.0001** |
|  |  |  |  | 1 vs 2: 0.03 |
|  |  |  |  | 1 vs 3: <0.0001 |
|  |  |  |  | 2 vs 3: <0.0001 |
| AJCC stage I/II | 76/102 (74.5%) | 34/68 (50.0%) | 58/111 (52.3%) | **0.0005** |
| AJCC stage III/IV | 26/102 (25.5%) | 34/68 (50.0%) | 53/111 (47.7%) |  |
|  |  |  |  | 1 vs 2: 0.002 |
|  |  |  |  | 1 vs 3: 0.0007 |
|  |  |  |  | 2 vs 3: 0.9 |
| Mucinous | 20/59 (33.9%) | 17/37 (45.9%) | 3/42 (7.1%) | **0.0003** |
|  |  |  |  | 1 vs 2: 0.3 |
|  |  |  |  | 1 vs 3: 0.002 |
|  |  |  |  | 2 vs 3: <0.0001 |
| Differentiation (poor) | 26/59 (44.1%) | 12/37 (32.4%) | 12/42 (28.6%) | 0.2 |
| CIMP (High) | 100/116 (86.2%) | 54/89 (60.7%) | 3/121 (2.5%) | **<0.0001** |
|  |  |  |  | 1 vs 2: <0.0001 |
|  |  |  |  | 1 vs 3: <0.0001 |
|  |  |  |  | 2 vs 3: <0.0001 |
| *p53* Mutation | 10/59 (16.9%) | 18/48 (37.5%) | 40/80 (50.0%) | **0.0002** |
|  |  |  |  | 1 vs 2: 0.03 |
|  |  |  |  | 1 vs 3: <0.0001 |
|  |  |  |  | 2 vs 3: 0.2 |
| *PRDM5* Methylation | 37/120 (30.8%) | 40/94 (42.6%) | 4/122 (3.3%) | **<0.0001** |
|  |  |  |  | 1 vs 2: 0.09 |
|  |  |  |  | 1 vs 3: <0.0001 |
|  |  |  |  | 2 vs 3: <0.0001 |
| *PRDM5* Average PMR | 21.2 | 29.5 | 3.3 | **<0.0001** |
|  |  |  |  | 1 vs 2: 0.05 |
|  |  |  |  | 1 vs 3: <0.0001 |
|  |  |  |  | 2 vs 3: <0.0001 |
| Nuclear Beta-Catenin | 25/59 (42.4%) | 11/33 (33.3%) | 38/44 (86.4%) | **<0.0001** |
|  |  |  |  | 1 vs 2: 0.5 |
|  |  |  |  | 1 vs 3: <0.0001 |
|  |  |  |  | 2 vs 3: <0.0001 |

Table S2: Clinical and Molecular Features of *BRAF* mutant cohorts stratified by *PRDM5* Methylation Status

|  | ***PRDM5* Status** | ***BRAF* mutant/MSI n=120** | ***BRAF* mutant/MSS n=94** | **P value between cohorts** |
| --- | --- | --- | --- | --- |
| Average Age | *PRDM5* Methylated  *PRDM5* Unmethylated | 74.6  77.8  P=0.07 | 70.0  69.9  P=0.8 | 0.09  **<0.0001** |
| Gender (female) | *PRDM5* Methylated  *PRDM5* Unmethylated | 27/37 (73.0%)  57/83 (68.7%)  P=0.7 | 21/40 (52.5%)  34/54 (63.0%)  P=0.4 | 0.1  0.6 |
| Location (proximal) | *PRDM5* Methylated  *PRDM5* Unmethylated | 31/35 (88.6%)  71/78 (91.0%)  P=0.7 | 31/37 (83.8%)  31/42 (73.8%)  P=0.4 | 0.7  **0.02** |
| AJCC  Stage I/II | *PRDM5* Methylated  *PRDM5* Unmethylated | 21/33 (63.6%)  55/69 (93.2%)  P=0.09 | 15/32 (46.9%)  19/36 (52.8%)  P=0.8 | 0.2  **0.007** |
| AJCC  Stage III/IV | *PRDM5* Methylated  *PRDM5* Unmethylated | 12/33 (36.4%)  14/69 (20.3%)  P=0.09 | 17/32 (53.1%)  17/36 (47.2%)  0.8 | 0.2  **0.007** |
| Mucinous | *PRDM5* Methylated  *PRDM5* Unmethylated | 3/21 (14.3%)  17/38 (44.7%)  P=**0.02** | 6/13 (46.2%)  11/24 (45.8%)  P=1.0 | 0.06  1.0 |
| Differentiation  (poor) | *PRDM5* Methylated  *PRDM5* Unmethylated | 10/21 (47.6%)  16/38 (42.1%)  P=0.8 | 5/13 (38.5%)  7/24 (29.2%)  P=0.7 | 0.7  0.4 |
| CIMP High | *PRDM5* Methylated  *PRDM5* Unmethylated | 32/37 (86.5%)  68/80 (85.0%)  P=1.0 | 27/36 (75.0%)  27/53 (50.9%)  P=**0.03** | 0.2  **<0.0001** |
| *p53* Mutation | *PRDM5* Methylated  *PRDM5* Unmethylated | 2/16 (12.5%)  8/43 (18.6%)  P=0.7 | 11/23 (47.8%)  7/25 (28.0%)  p=0.2 | **0.04**  0.4 |
| Nuclear Beta-Catenin | *PRDM5* Methylated  *PRDM5* Unmethylated | 13/21 (61.9%)  12/38 (31.6%)  **p=0.03** | 3/12 (25.0%)  8/21 (38.1%)  p=0.7 | 0.07  0.8 |

Table S3: Clinical data for serrated polyp and conventional adenoma cohorts

|  | Serrated Polyps | | | Conventional Adenomas | |
| --- | --- | --- | --- | --- | --- |
|  | MVHP | SSA | TSA | TA | TVA |
| n | 19 | 20 | 20 | 20 | 20 |
| Age (years) | 52.1 | 60.5 | 63.9 | 60.1 | 57.4 |
| Gender - Female | 9 (47.3%) | 10 (50.0%) | 11 (55.0%) | 10 (50.0%) | 7 (35.0%) |
| Size (<10mm) | 18 (94.7%) | 14 (70.0%) | 4 (20.0%) | 17 (85.0%) | 4 (20.0%) |
| Location - proximal | 7 (36.8%) | 10 (50.0%) | 4 (20.0%) | 10 (50.0%) | 2 (10.0%) |
| Dysplasia present | 0 | 0 | 3 (15.0%) | high grade:  0 | high grade:  2 (10.0%) |
